# Studies on heavy metal removal efficiency and antibacterial activity of chitosan prepared from shrimp shell waste

**DOI:** 10.1007/s13205-013-0140-6

**Published:** 2013-05-26

**Authors:** V. Mohanasrinivasan, Mudit Mishra, Jeny Singh Paliwal, Suneet Kr. Singh, E. Selvarajan, V. Suganthi, C. Subathra Devi

**Affiliations:** School of Biosciences and Technology, VIT University, Vellore, 14 Tamil Nadu India

**Keywords:** Shrimp shells, Chitosan, Deacetylation, Metal removal efficiency, Antibacterial activity

## Abstract

Chitosan, a natural biopolymer composed of a linear polysaccharide of α (1–4)-linked 2-amino 2-deoxy β-d glucopyranose was synthesized by deacetylation of chitin, which is one of the major structural elements, that forms the exoskeleton of crustacean shrimps. The present study was undertaken to prepare chitosan from shrimp shell waste. The physiochemical properties like degree of deacetylation (74.82 %), ash content (2.28 %), and yield (17 %) of prepared chitosan indicated that that shrimp shell waste is a good source of chitosan. Functional property like water-binding capacity (1,136 %) and fat-binding capacity (772 %) of prepared chitosan are in total concurrence with commercially available chitosan. Fourier Transform Infra Red spectrum shows characteristic peaks of amide at 1,629.85 cm^−1^ and hydroxyl at 3,450.65 cm^−1^. X-ray diffraction pattern was employed to characterize the crystallinity of prepared chitosan and it indicated two characteristic peaks at 10° and 20° at (2*θ*). Scanning electron microscopy analysis was performed to determine the surface morphology. Heavy metal removal efficiency of prepared chitosan was determined using atomic absorption spectrophotometer. Chitosan was found to be effective in removing metal ions Cu(II), Zn(II), Fe(II) and Cr(IV) from industrial effluent. Antibacterial activity of the prepared chitosan was also determined against *Xanthomonas* sp. isolated from leaves affected with citrus canker.

## Introduction

**Fig. 1:**
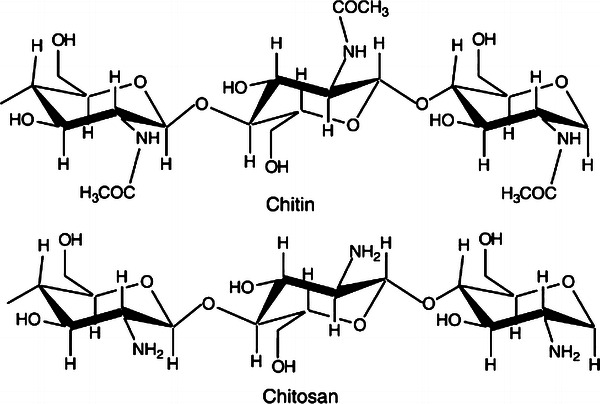
Showing the molecular structure of chitosan (Jayakumar et al. 2011)

Chitosan is one of the most important derivatives of chitin, which is the second most abundant natural biopolymer found on earth after cellulose (No and Meyers 1989) and is a major component of the shells of crustaceans such as crabs and shrimps. Chitosan can be obtained by *N*-deacetylation of chitin and it is a co-polymer of glucosamine and *N*-acetylglucosamine units linked by 1–4 glucosidic bonds (Fig. 1). Chitosan is a fiber-like cellulose only but unlike plant fibers, it possesses some unique properties including the ability to form films, optical structural characteristics, and much more. Chitosan have the ability to chemically bind with negatively charged fats, lipids, and bile acids and this ability is because of the presence of a positive ionic charge (Sandford 1992). In acidic conditions (pH < 6), chitosan becomes water soluble that enables the formation of biocompatible and very often biodegradable polymers with optimized properties in homogenous solutions. Chitosan being a non-toxic, biodegradable, and biocompatible polysaccharide polymer have received enormous worldwide attention as one of the promising renewable polymeric materials for their extensive applications in industrial and biomedical areas such as paper production, textile finishes, photographic products, cements, heavy metal chelation, waste water treatment fiber, and film formations (Rathke and Hudson 1994). It can also be used in biomedical industries for enzyme immobilization and purification, in chemical plants for wastewater treatment, and in food industries for food formulations as binding, gelling, thickening, and stabilizing agent (Knorr 1984).

As chitosan can be readily converted into fibers, films, coatings, and beads as well as powders and solutions, further enhance its applications. The functional properties of chitosan are dependent on its molecular weight and its viscosity (No and Lee 1995). The presence of both free hydroxyl and amine groups enables chitosan to be modified readily to prepare different chitosan derivatives (Kurita 2001) that give some sophisticated functional polymers with exquisite properties quite different from those of the synthetic polymers. With its positive charge, chitosan can be used for coagulation and recovery of proteinaceous materials present in food processing operations (Knorr 1991). Chitosan has largely been employed as a non-toxic flocculent in the treatment of organic polluted wastewater and as a chelating agent for the removal of toxic (heavy and reactive) metals from industrial wastewater (An et al. 2001). There are some metals which exist in aqueous solutions as anions like chromium (Rhazi et al. 2002). Chitin can be effectively extracted from prawn shells following deprotienization using 5 % NaOH and demineralization using 1 % HCl. Low molecular mass chitosan samples with degree of deacetylation (DD) >64 % and Mw of the major component <104 can be obtained by treating the chitin with 50 % NaOH at 100 °C for up to 10 h (Mohammed et al. 2012). At pH close to neutral the amine groups of chitosan binds to metal cations. At lower pH it is able to bind more of anions by electrostatic attraction as chitosan gets more protonated (Guibal 2004). Chitosan can be readily used as a biosorbent as it is cheaply available cationic biopolymer. Chitosan has antimicrobial activity, haemostatic activity, anti-tumor activity, accelerates wound healing, can be used tissue-engineering scaffolds and also for drug delivery (Burkatovskaya et al. 2006). The antimicrobial activity and antifungal activity of chitosan is largely because of its polycationic nature (Ziani et al. 2009 and Choi et al. 2001). It displays broad spectrum of antibacterial activity against both gram positive and gram negative bacteria and also antifungal activity against *Aspergillus niger*, *Alternaria alternata*, *Rhizopus oryzae*, *Phomopsis asparagi*, and *Rhizopus stolonifer* (Guerra-Sánchez et al. 2009; Zhong et al. 2007; Ziani et al. 2009). Chitin exhibited a bacteriostatic effect on gram-negative bacteria, *Escherichia coli* ATCC 25922, *Vibrio cholerae*, *Shigella dysenteriae,* and *Bacteroides fragilis* (Benhabiles et al. 2012).

The current research is to prepare chitosan from shrimp shell waste and to estimate the prepared chitosan by both qualitatively and quantitatively. Other applications such as antibacterial activity against *Xanthomonas* sp. and metal removal efficiency have also been studied for the prepared chitosan.

## Materials and methods

### Materials

Shrimps shell waste material was collected from local market of Vellore. The chemicals such as Hydrochloric acid and Sodium hydroxide pellets were procured from Hi-Media Laboratory, Mumbai. Distilled water was used throughout the process.

### Preparation of chitosan

**Fig. 2:**
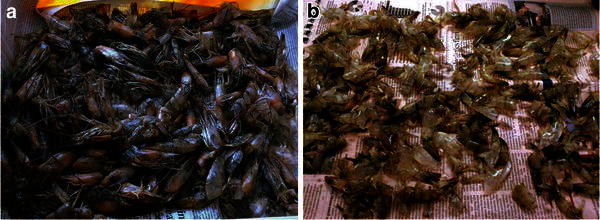
**a** Shrimp shell waste. **b** Shrimp shells after washing and drying

**Fig. 3:**
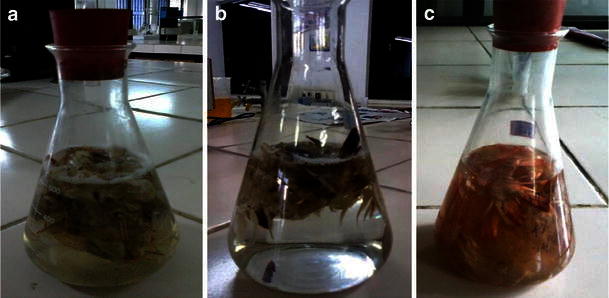
Flasks showing shrimp shells during **a** demineralization, **b** deprotienization, and **c** deacetylation steps

**Fig. 4:**
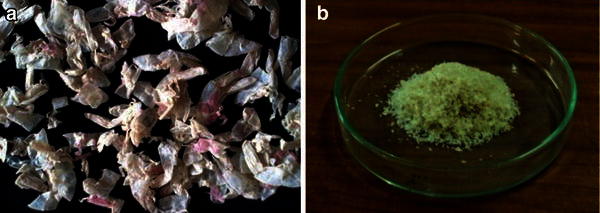
Prepared chitosan **a** before grinding and **b** chitosan flakes after grinding

The shrimp shells obtained from the local market of Vellore shown in Fig. 2a were first suspended in 4 % HCl at room temperature in the ratio of 1:14 (w/v) for 36 h. This causes the Demineralization of shells after which they were washed with water to remove acid and calcium chloride. Deproteinization of shells was done by treating the demineralized shells with 5 % NaOH at 90 °C for 24 h with a solvent to solid ratio of 12:1 (v/w). After the incubation time the shells were washed to neutrality in running tap water and sun dried. The product obtained was chitin. Chitosan preparation involves the deacetlyation of the obtained chitin (Dutta et al. 2004). Deacetylation of chitin involves the removal of acetyl groups from chitin and that was done by employing 70 % NaOH solution with a solid to solvent ratio of 1:14 (w/v) and incubated at room temperature for 72 h. Stirring is mandatory to obtain a homogenous reaction as shown in Fig. 3a–c. The residue obtained after 72 h was washed with running tap water to neutrality and rinsed with deionized water. It was then filtered, sun dried and finely grinded shown in Fig. 4a. The resultant whitish flakes obtained after grinding are chitosan as shown in Fig. 4b.

### Determination of chitosan yield

Yield was determined by taking the dry weight of shrimp shells before treatment and the dry weight of prepared chitosan.

### Determination of ash content

The ash content of the prepared chitosan was determined by placing 0.5706 g of chitosan into previously ignited, cooled, and tarred crucible. The samples were heated in a muffle furnace preheated to 600 °C for 6 h. The crucibles were allowed to cool in the furnace to <200 °C and then placed into desiccators with a vented top. Crucible and ash was weighed (AOAC 1990).

### Determination of moisture content

Moisture content was determined by employing the gravimetric method (Black 1965). The water mass was determined by drying the sample to constant weight and measuring the sample after and before drying. The water mass (or weight) was the difference between the weights of the wet and oven dry samples. Then moisture content was calculated using the following relationship:

### Determination of solubility

The solubility of prepared chitosan was determined by taking 200 mg of chitosan and then adding 200 ml of water and the same method was followed with 1 % acetic acid solution.

### FTIR analysis

The samples of prepared chitosan were characterized in KBr pellets by using an infrared spectrophotometer in the range of 400–4,000 cm^−1^.

### Degree of deacetylation

The DD of chitosan was determined using a Fourier Transform Infra Red (FTIR) instrument with frequency of 4,000–400 cm^−1^. The following equation (Struszczyk 1987) was used, where the absorbance at A1629.85 and A3450.65 cm^−1^ are the absolute heights of absorption bands of amide and hydroxyl groups, respectively.

The factor ‘1.33’ denoted the value of the ratio of A1629.85/A3450.65 for fully *N*-acetylated chitosan.

### Determination of WBC

This property of chitosan was determined by using the modified method of (Wang and Kinsella 1976). For water-binding capacity (WBC) 0.5 g of chitosan sample was taken in a centrifuge tube of 50 ml which was weighed initially and then adding 10 ml of water, and mixing on a vortex mixer for 1 min to disperse the sample. The contents were later left at ambient temperature for 30 min with intermittent shaking for 5 s every 10 min and then centrifuged at 3,200 rpm for 25 min. After centrifugation the supernatant was decanted and the tube was weighed again and WBC was calculated using the following relationship.

### Determination of FBC

Fat-binding capacity of prepared chitosan was calculated using the modified equation of (Wang and Kinsella 1976). For FBC 0.5 g of chitosan sample was taken in a 50 ml centrifuge tube which was weighed initially and 10 ml of gingelly oil was taken followed by mixing on a vortex mixer for 1 min to disperse the sample. The contents were later left at ambient temperature for 30 min with intermittent shaking for 5 s every 10 min and then centrifuged at 3,200 rpm for 25 min. After the centrifugation the supernatant was decanted and the tube was weighed again and FBC was calculated using the following relationship.

### XRD analysis

The prepared chitosan was characterized by X-ray diffraction (XRD) technique using an X-ray diffractometer (Bruker Germany, D8 Advance, 2.2 KW Cu Anode, Ceramic X-ray) with CuKα radiation (*λ* = 1.5406 Ǻ). The measurement was in the scanning range of 5–70 at a scanning speed of 50 s^−1^.

### SEM analysis

Chitosan prepared from shrimp shell waste was examined by scanning electron microscopy (SEM) having a magnification range of 5,000 and accelerating voltage 20 kV.

### Determination of heavy metals removal efficiency in industrial effluents by AAS

0.1 g of chitosan was mixed with 40 ml of industrial effluent (obtained from leather industry located at Ranipet) and its pH was measured, followed by an incubation of 3 h at 22 °C. The original effluent was used as control. The industrial effluent mixed with chitosan was kept for incubation for 3 h at 22 °C. The contents were then centrifuged at 7,000 rpm (revolutions per minute) for 5 min, and supernatant was filtered using Whatman filter paper no 2. The metal ions namely Cr(IV), Fe(II), Zn(II) and Cu(II) were analyzed for their residual metal concentration using atomic absorption spectrophotometer (AAS) (VARIAN, AA240). The standards of these metals were prepared (Gamage and Shahidi 2007).

### Determination of inhibitory activity of chitosan against *Xanthomonas* sp.

Leaves showing symptoms of cankerous growth were plucked from lemon tree (*Citrus limon*) and were surface sterilized with sodium sulfite for consecutively seven times followed by distilled water. Leaves were crushed in mortar and pestle with 1 ml deionized water and the extract obtained was spread on nutrient agar plates supplemented with 5 % sucrose.

Method employed for evaluating the antimicrobial activity was growth inhibition in liquid medium. The antimicrobial effect of prepared chitosan was studied in liquid nutrient medium. Nutrient broth supplemented with 5 % sucrose was used. The flasks were marked as blank that contained only the media, control (standard) that had the bacterial culture only and the test which contained the prepared chitosan and *Xanthomonas* sp. cultures. The freshly grown inoculums were allowed to incubate in the presence of 0.2 g of chitosan to observe the bacterial growth pattern at 310 K (37 °C) and 150 rpm. In liquid medium, growth of *Xanthomonas* sp. was indexed by measuring the optical density (OD). Optical density measurements were carried out at *λ*_max_ = 600 nm after every 1 h interval up to 24 h. Graph was plotted to interpret the results.

## Results and discussion

### Yield

The prepared chitosan had a percentage yield of 17 % as shown in Table 1, which was at par when compared to the percentage yield obtained by (Brzeski 1982) who reported 14 % yield of chitosan from krill and showed no significant difference to the percentage yield of 18.6 % from prawn waste (Alimuniar and Zainuddin 1992).

**Table 1:** Physiochemical and functional properties of chitosan

Properties	Percentage
Yield	17
Moisture content	1.25
Ash	2.28
DD	74.82
WBC	1,136
FBC	772
Solubility	1 % CH_3_COOH

### Ash content

The prepared chitosan had an ash content of 2.28 % as shown in Table 1, which when compared to commercial chitosan which had an ash content of 2 % shows that the chitosan prepared had a standard percentage of ash content which can be used for commercial applications, as the ash content in chitosan is an important parameter that affects its solubility, viscosity and also other important characteristics.

### Moisture content

The moisture content of chitosan obtained from shrimp shells was measured to be 1.25 % as shown in Table 1, which is in agreement with (Islam et al. 2011 and Hossein et al. 2008) who reported moisture content in the range of 1–1.30 obtained from brine shrimp shells. Although Li et al. (1992) reported that commercial chitosan products may contain <10 % moisture content.

### Solubility

The prepared chitosan from shrimp shells waste was found to be soluble in 1 % acetic acid solution and partially soluble in water as shown in Table 1.

### Degree of deacetylation

In the present study DD of the prepared chitosan was found to be 74.82 % (Table 1). It is an important parameter that influences other properties like solubility, chemical reactivity and biodegradability. DD of the commercially available chitosan has values that range between 75 and 85 %. The value of DD depends on various factors such as the source and procedure of preparation and the values ranges from 30 to 95 % (Martino et al. 2005) and also on the type of analytical methods employed, sample preparation, type of instruments used, and other conditions may also influence the analysis of DD (Khan et al. 2002).

### WBC and FBC

Water-binding capacity and FBC are functional properties that vary with the method of preparation. Chitosan prepared from shrimp shells in the present study has WBC and FBC of 1,136 and 772 % (Table 1) and these are in agreement with studies reported by (Rout 2001).

### XRD analysis

**Fig. 5:**
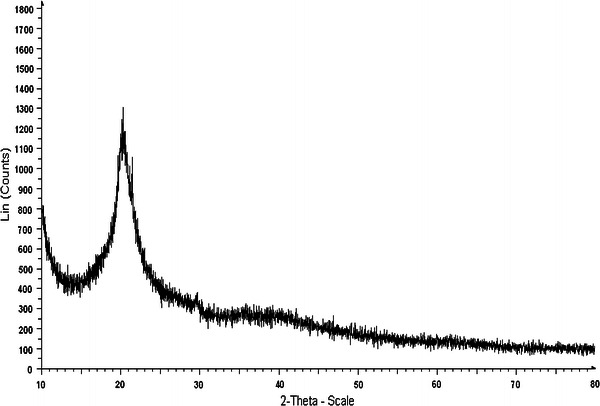
XRD of prepared chitosan

**Fig. 6:**
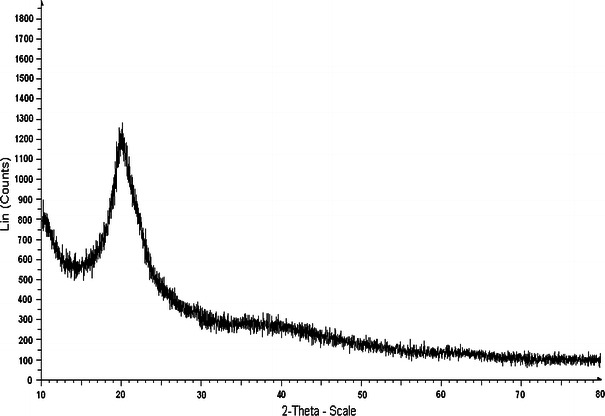
XRD of standard chitosan

The XRD pattern of chitosan prepared from shrimp shells waste illustrates two characteristic broad diffraction peaks at (2*θ*) = 10° and 20° that are typical fingerprints of semi-crystalline chitosan as shown in Fig. 5 (Bangyekan et al. 2006). The XRD pattern of standard chitosan procured from Sigma Aldrich shows similar peaks as shown in Fig. 6. The peaks around 2*θ* = 10° and 2*θ* = 20° are related to crystal I and crystal II in chitosan structure (Ebru et al. 2007; Marguerite 2006) and both these peaks attributes a high degree of crystallinity to the prepared chitosan (Julkapli and Akil 2007) as shown in Fig. 5.

### FTIR analysis

**Fig. 7:**
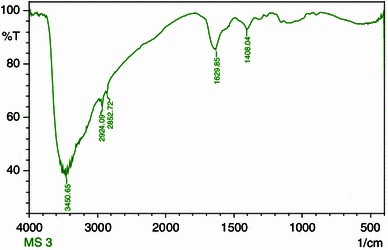
FTIR of prepared chitosan

The structure of the prepared chitosan was confirmed by FTIR analysis. The spectra of chitosan shows a broad absorption band in the region of 3,450.65 cm^−1^ that corresponds to OH stretching vibrations of water and hydroxyls and NH stretching vibrations of free amino groups as shown in Fig. 7. The band observed at 2,924.09 and 2,852.72 corresponds to asymmetric stretching of CH_3_ and CH_2_ in the prepared chitosan (Guo et al. 2005). The intensive peak around 1,629.85 cm^−1^ corresponds to bending vibration of NH_2_ which is a characteristic feature of chitosan polysaccharide and also indicates the occurrence of deacetylation (Zhang et al. 2011 and Radhakumary et al. 2003).

### SEM analysis

**Fig. 8:**
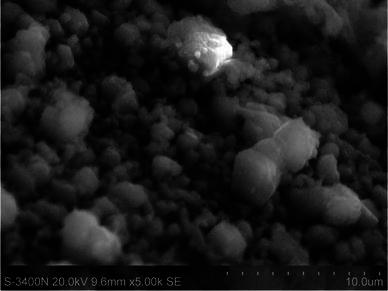
SEM image of prepared chitosan

The SEM micrograph illustrates the morphology of the prepared chitosan from shrimp shells. The micrographs showed non-homogenous and non-smooth surface as shown in Fig. 8.

### Heavy metals removal efficiency in industrial effluents by AAS

**Fig. 9:**
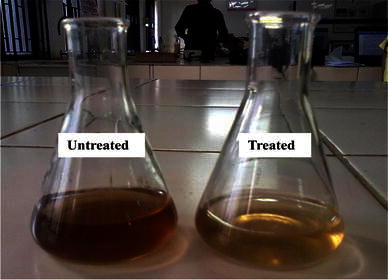
The untreated and treated effluents showing change in color before and after treatment with chitosan

Industrial effluent obtained from Ranipet region contained traces of heavy metals namely Cu(II), Zn(II), Fe(II) and Cr(IV) that was confirmed by AAS. The results indicated that the prepared chitosan has the ability to adsorb the metal ions that were present in the industrial effluent as shown in Table 2. Out of all the metal ions Cu(II) was best absorbed showing removal of 98.97 %. Zinc and its salts have high acute and chronic toxicity to aquatic life in polluted water. Zinc toxicity causes problems like nausea, vomiting, diarrhea and also sometimes abdominal cramps (Elinder and Piscator 1979). Zinc present in the industrial effluent was successfully absorbed by the prepared chitosan to around 86.15 % AAS results strongly indicates the removal of metal ions where the sample 1 used is the untreated one and sample 2 is the treated one. Fe(II) which is responsible for the unpleasant organoleptic properties in drinking water (Muzzzarelli et al. 1989) has also been absorbed by the prepared chitosan to less percentage of around 65.2 %. The efficacy of chitosan treated and untreated effulents were shown in Fig. 9. Therefore it can be concluded that the prepared chitosan has the potential to be used as a adsorbent in the treatment of industrial wastewater.

**Table 2:** Heavy metal removal percentage

Metal	Removal (%)
Cu(II)	98.97
Cr(IV)	37.51
Fe(II)	65.2
Zn(II)	86.15

*Cu* copper, *Cr* chromium, *Fe* Iron, *Zn* zinc

### Inhibitory activity of chitosan on *Xanthomonas* sp.

**Fig. 10:**
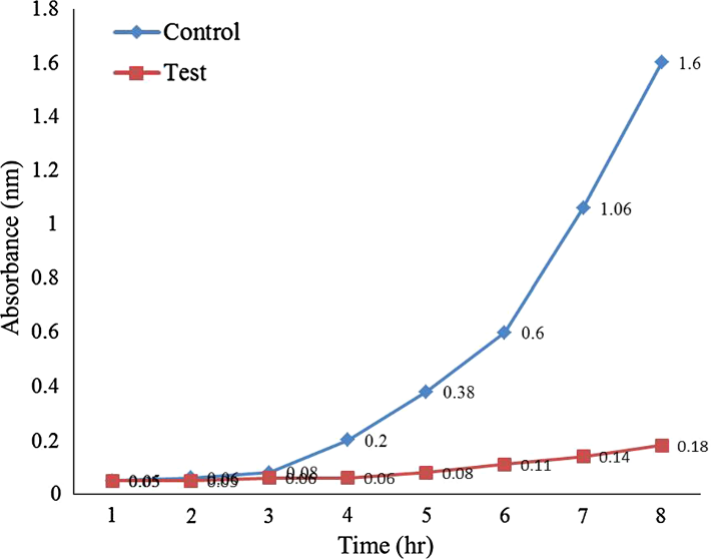
Graph showing inhibitory activity of chitosan on *Xanthomonas* sp.

Optical density (OD) measurements were indexed and turbidity was observed to evaluate the inhibitory activity of chitosan. Growth of *Xanthomonas* sp. was inhibited by the chitosan in the liquid medium. Very less turbidity was there in the test flask which contained both the chitosan and the organism. Whereas, the standard (control) that had only the organism was turbid and by the increase in OD readings showed that there was growth. Graph was plotted to determine the difference in the growth pattern of test ant control as shown in Fig. 10. No activity was observed in the blank.

## Conclusion

The present study observations indicate that chitosan has been successfully prepared from shrimp shell waste. The functional, physiochemical properties, XRD and FTIR of the prepared chitosan showed that it can be used commercially and can be supplemented in food and drug preparation. The prepared chitosan was found effective in removing metals from industrial effluent and the result clearly indicated that the metal ion percentage was reduced to mere negligible level. Inhibition in growth of *Xanthomonas* sp. was observed in presence of chitosan prepared from shrimp shells. Since chitosan has the potential to be used as an antibacterial agent to control plant diseases. Method employed in this study prepares chitosan in a very economical way and it can also be a way to control pollution as shrimp shell waste is being used which is otherwise discarded.

## Abbreviations

DD: Degree of deacetylation
WBC: Water-binding capacity
FBC: Fat-binding capacity
FTIR: Fourier transform infrared
XRD: X-ray diffraction
SEM: Scanning electron microscopy
AAS: Atomic absorption spectroscopy
OD: Optical density
